# Delirium Management Quality Improvement Project to Improve Awareness and Screening in a Medical ICU

**DOI:** 10.3390/nursrep15010006

**Published:** 2024-12-30

**Authors:** Hirsh Makhija, Kyle Digrande, Omar Awan, Russell G. Buhr, Rajan Saggar, Victoria Ramirez, Rainbow Tarumoto, Janelle M. Fine, Atul Malhotra, Dale M. Needham, Jennifer L. Martin, Biren B. Kamdar

**Affiliations:** 1 Division of Pulmonary, Critical Care and Sleep Medicine, UC San Diego Health, La Jolla, CA 92093, USA; himakhij@ucsd.edu (H.M.); jfine@health.ucsd.edu (J.M.F.); amalhotra@health.ucsd.edu (A.M.); 2 Department of Medicine, University of California, Irvine, CA 92697, USA; kdigrand@hs.uci.edu; 3 Pulmonary Section, Medicine Service, VA Medical Center, Washington, DC 20422, USA; omar.awan@va.gov; 4 Department of Medicine, George Washington University, Washington, DC 20052, USA; 5 Division of Pulmonary, Critical Care and Sleep Medicine, David Geffen School of Medicine, University of California, Los Angeles, CA 90095, USA; rbuhr@mednet.ucla.edu (R.G.B.); rsaggar@mednet.ucla.edu (R.S.); 6 Center for the Study of Healthcare Innovation, Implementation, and Policy, Health Services Research & Development, VA Greater Los Angeles Healthcare System, Los Angeles, CA 90073, USA; 7 Medical Intensive Care Unit, Ronald Reagan UCLA Hospital, Los Angeles, CA 90095, USA; vickyccrn@att.net; 8 Division of Trauma, Surgical Critical Care and Acute Care Surgery, Department of Surgery, Harbor-UCLA Medical Center, Torrance, CA 90509, USA; rtarumoto@ucdavis.edu; 9 Division of Pulmonary and Critical Care Medicine, Johns Hopkins University School of Medicine, Baltimore, MD 21205, USA; dale.needham@jhmi.edu; 10 Department of Physical Medicine and Rehabilitation, Johns Hopkins University School of Medicine, Baltimore, MD 21205, USA; 11 School of Nursing, Johns Hopkins University, Baltimore, MD 21205, USA; 12 VA Greater Los Angeles Healthcare System, Geriatric Research, Education and Clinical Center, Los Angeles, CA 90073, USA; jennifer.martin@va.gov; 13 Department of Medicine, David Geffen School of Medicine, University of California, Los Angeles, CA 90095, USA

**Keywords:** benzodiazepines, delirium, intensive care unit, outcome assessment, quality improvement, nursing

## Abstract

**Background/Objectives:** Although delirium is common during critical illness, standard-of-care detection and prevention practices in real-world intensive care unit (ICU) settings remain inconsistent, often due to a lack of provider education. Despite availability for over 20 years of validated delirium screening tools such as the Confusion Assessment Method in the ICU (CAM-ICU), feasible and rigorous educational efforts continue to be needed to address persistent delirium standard-of-care practice gaps. **Methods:** Spanning an 8-month quality improvement project period, our single-ICU interdisciplinary effort involved delivery of CAM-ICU pocket cards to bedside nurses, and lectures by experienced champions that included a live delirium detection demonstration using the CAM-ICU, and a comprehensive discussion of evidence-based delirium prevention strategies (e.g., benzodiazepine avoidance). Subsequent engagement by health system leadership motivated the development of an electronic health record dataset to evaluate unit-level outcomes, including CAM-ICU documentation and benzodiazepine administration. **Results:** Using a dataset that spanned 9 pre- and 37 post-project months and included 3612 patients, 4470 admissions, and 33,913 patient days, we observed that delirium education was followed by a dramatic rise in CAM-ICU documentation, from <1% for daytime and nighttime shifts to peaks of 73% and 71%, respectively (*p* < 0.0001 for trend), and a fall in the proportion of mechanically ventilated patients ever receiving benzodiazepine infusions (69% to 41%; *p* < 0.0001). **Conclusions:** An interdisciplinary delirium project comprising rigorous lectures on standard-of-care practices can yield significant improvements in documentation and sedative administration. This approach can help ICUs jumpstart efforts to build awareness and address longstanding gaps in standard-of-care delirium practices.

## 1. Background

Delirium is a common and preventable syndrome associated with a variety of adverse short- and long-term outcomes, including prolonged intensive care unit (ICU) and hospital length of stay [1,2]. Affecting ~40% of ICU and up to ~80% of mechanically ventilated patients, delirium is costly, accounting for approximately $150 billion in annual healthcare costs in the US alone [2,3,4].

While increasing awareness has motivated delirium prevention efforts (i.e., sedation and mobility protocols), essential to these efforts are early, accurate, and sustained delirium detection. In their Clinical Practice Guidelines for Pain, Agitation, and Delirium (PAD; published in 2013) and PAD, Immobility, and Sleep Disruption (PADIS; published in 2018) in the ICU, the Society of Critical Care Medicine (SCCM) recommended that patients be monitored daily for delirium using either the confusion assessment method for the ICU (CAM-ICU) or the Intensive Care Delirium Screening Checklist (ICDSC) [5,6,7]. Despite these recommendations and the availability of these validated, free, and easy-to-administer tools, many ICUs struggle to consistently perform daily delirium screening, rendering patients vulnerable to adverse outcomes related to delirium under-detection [3,8]. Specifically, studies over the past six years have shown screening levels as low as 31%, with some ICUs having a complete absence of formal screening education and protocols [9,10,11,12].

In March 2013, a single academic MICU began requiring daytime and nighttime nursing staff to document CAM-ICU assessments at least once per shift. Despite this requirement, staff reported inconsistent bedside CAM-ICU documentation practices, citing various barriers including the absence of champions and inadequate delirium education. In this manuscript, we present the results of a simple, grass-roots interdisciplinary effort aimed at optimizing delirium awareness and documentation, along with delirium-related sedation practices [8,13,14,15].

## 2. Materials and Methods

This quality improvement initiative took place from November 2013 to December 2016 in a single academic MICU within the United States, which featured 24 private rooms and a nurse-to-patient ratio of 1 to 2, with nurses working either daytime (7 a.m. to 7 p.m.) or nighttime (7 p.m. to 7 a.m.) shifts. This analysis was reported in accordance with SQUIRE 2.0 guidelines [16].

### 2.1. Project Timeline

**Figure 1:**
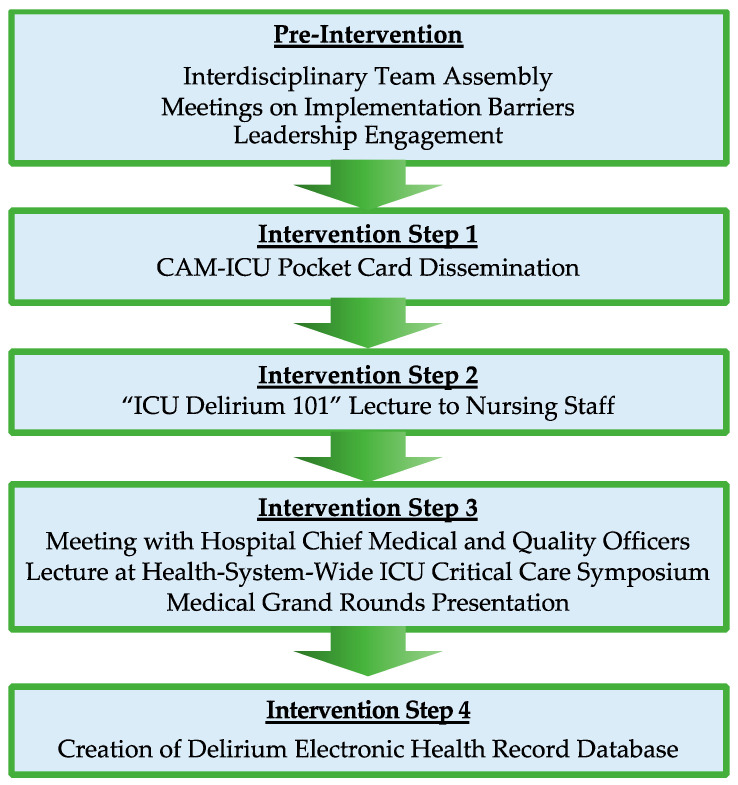
Interdisciplinary delirium quality improvement project conceptual framework.

Motivated by prior ICU delirium efforts, this quality improvement project was jumpstarted by bedside conversations and interactions that highlighted staff knowledge gaps regarding delirium following the March 2013 introduction of a required once-per-shift CAM-ICU flowsheet in the electronic health record (EHR) [17,18]. These interactions and conversations highlighted a critical need for delirium education, thus motivating the assembly of an interdisciplinary team (including a nurse who experienced delirium as a patient in the ICU, the MICU director, a physician champion, and stakeholders from nursing leadership, rehabilitation services, and respiratory therapy) tasked with heightening ICU provider awareness and formalizing daily delirium screening in the MICU (Figure 1).

Through three meetings over three months, the interdisciplinary team outlined local implementation barriers, which included a lack of local delirium educational tools upon which to build, a large number of bedside nurses who would need education, and stakeholder time constraints that would make large-scale one-on-one education infeasible. Given these challenges, the team initiated the project by producing and distributing laminated CAM-ICU pocket cards among ICU nursing staff in late 2013 and early 2014. When possible, the distribution of pocket cards was accompanied by nudges from champions and bedside nurses to complete EHR CAM-ICU documentation at least once per shift. In February 2014, the physician champion arranged for a visiting ICU outcomes expert to give a talk to ICU nurses on post-intensive care syndrome, a lecture that included information about outcomes related to ICU delirium. With prompting of nursing leadership, in July 2014, the physician champion developed and delivered an “ICU Delirium 101” lecture to MICU nursing staff during their lunch break. This 45 min lecture included an overview of ICU delirium, including its definition, associated risk factors and outcomes, a live CAM-ICU demonstration emphasizing the importance of daily delirium screening, and, finally, simple strategies to prevent delirium (i.e., sedation minimization) (Table 1). Following positive feedback from the first lecture, the physician champion repeated the lecture for night shift nursing staff.

### 2.2. Systems-Based Innovations

As delirium awareness rose locally within the ICU, the physician champion was invited to share selected lecture slides with the hospital Chief Medical (CMO) and Quality Officers (CQO), emphasizing the high prevalence and cost of delirium and its impact on patient outcomes. This meeting led to acknowledgment of delirium as a system-wide priority, stimulating the invitation of the delirium champion to lecture on delirium at a health system-wide ICU Critical Care Symposium in March 2015, Medical Grand Rounds in May 2015, and with physician trainees and nurses from other ICUs (multiple occasions). In May 2015, the Office of Health Informatics and Analytics (OHIA) approved the creation of an EHR-based delirium database, which was finalized in September 2015 and included CAM-ICU data and delirium-associated variables (e.g., sedative use). Queried data included all values within pre-specified fields from every ICU patient’s EHR, yielding a dataset whose missing values were only those that were left blank (e.g., not documented by staff). These data were presented by the physician champion to an interdisciplinary audience at the Pulmonary and Critical Care Grand Rounds in March 2016. Analyst availability for dataset updates was provided until 31 December 2016, which included the latest information on sedatives ordered by physicians and titration controlled by bedside nurses.

### 2.3. Data Collection and Analysis

The primary outcome was monthly delirium documentation rates, calculated as the proportion of potential CAM-ICU daytime and nighttime assessments documented in the electronic health record (EHR). The secondary outcomes analyzed were whether ICU patients ever received a sedative infusion (midazolam, fentanyl, dexmedetomidine, propofol) and the cumulative dosing of these drugs.

The delirium dataset included demographic and clinical data for every patient admitted to the ICU from EHR inception to the end of the improvement period. Clinical data included admission diagnosis category, mechanical ventilation status, and administration of sedative infusions. Notably, during the data collection and project period, there were no other competing projects, or changes in MICU staffing or admission trends. Additionally, as part of ongoing efforts in our medical ICU, the institutional review board deemed this project as quality improvement and therefore exempt from full review.

We used Poisson regression to evaluate temporal trends in the monthly incidence of CAM-ICU documentation and sedative infusion practices. Negative binomial regression was used to evaluate monthly trends in sedative dosing. Stata version 15.0 (StataCorp, College Station, TX, USA) was used for all analyses [19].

## 3. Results

From 1 March 2013 (the inception of the CAM-ICU flowsheet in the EHR) to 31 December 2016, 3612 patients were admitted to the MICU; 45% were female and 37% non-White, with a median (IQR) age of 62 (49, 73) years (Table 2). Across 4470 ICU admissions, the median (IQR) ICU length of stay was 5 (3, 8) days, and 36% involved mechanical ventilation for a median (IQR) duration of 3 (2, 7) days, with fentanyl (66%), midazolam (52%), propofol (32%), and dexmedetomidine (19%) comprising the most common sedatives ever infused during those admissions (Table 2).

### 3.1. Delirium Documentation

**Figure 2:**
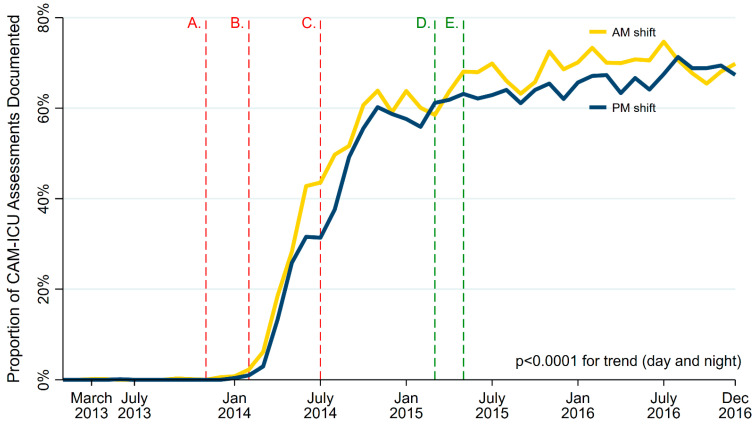
Monthly proportion of CAM-ICU assessments documented at least once per daytime and nighttime shift per patient. Vertical lines highlight ICU- (red) and health system-wide (green) innovations: A. Distribution of CAM-ICU cards to nursing staff; B. Lecture on Post-intensive Care Syndrome for ICU Providers; C. ‘ICU Delirium 101’ lecture for MICU nurses; D. ‘ICU Delirium 101’ lecture at UCLA Critical Care Symposium; E. UCLA Office of Health Informatics and Analytics (OHIA) approves development of EHR-based dataset to evaluate CAM-ICU and other delirium metrics. The *p*-value was calculated using Poisson regression, including CAM-ICU documentation data from all ICU admits.

From March to December 2013, 0.07% (10 out of 14,578) of potential CAM-ICU assessments were documented in the EHR. In November 2014, CAM-ICU documentation rates rose, peaking at 64% during daytime and 60% during nighttime shifts and plateauing at 73% and 65%, respectively, in late 2015 (daytime and nighttime month-to-month IRR from March 2013 to December 2016 = 1.05 and 1.05, respectively; each *p* < 0.0001 for trend; Figure 1). In mechanically ventilated patients, CAM-ICU documentation rates rose from 0% in 2013 to 66% during daytime and nighttime shifts in late 2014, plateauing at 75% and 70%, respectively, in late 2015 (daytime and nighttime IRR = 1.05 and 1.05, respectively; each *p* < 0.0001; Figure 2).

### 3.2. CAM-ICU Scoring

Following 2013, the proportion of “unable to assess” scores varied over time. Specifically, among CAM-ICUs documented during daytime shifts from August 2014 (1 month after the “ICU 101 lecture”) to December 2016, the proportion of “unable-to-assess” ratings ranged from 15% to 44% (median [IQR] = 27% [22%, 30%]). Across nighttime CAM-ICU assessments, the proportion of “unable-to-assess” ratings ranged from 15% to 49% (median [IQR] = 27% [23%, 31%]).

In mechanically ventilated patients with documented daytime CAM-ICUs, from August 2014 to December 2016, the proportion of “unable-to-assess” ratings ranged from 23% to 64% (median [IQR] = 43% [35%, 48%]). Across nighttime CAM-ICU assessments, the proportion of “unable-to-assess” ratings ranged from 25% to 68% (median [IQR] = 44% [37%, 49%]).

### 3.3. Delirium Prevalence

The proportion of patients with a positive CAM-ICU assessment for delirium was unknown until EHR inception in March 2013. The proportion of monthly admissions with positive delirium assessments on ICU admission peaked at 0% in 2013, 13% in 2014, 15% in 2015, and 10% in 2016. The proportion of monthly admissions with a positive assessment following a negative delirium assessment at ICU admission peaked at 1% in late 2013, 17% in 2014, 22% in 2015, and 18% in 2016. Monthly delirium prevalence peaked at 1% in late 2013, 28% in 2014, 32% in 2015, and 25% in 2016.

### 3.4. Sedative Infusions

**Figure 3:**
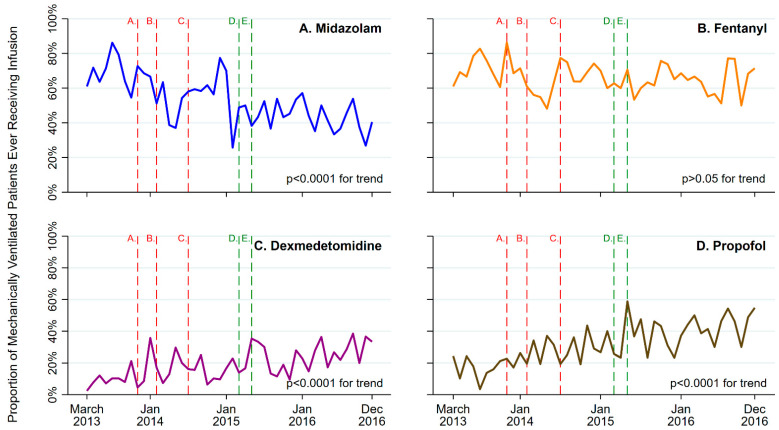
Monthly proportion of mechanically ventilated patients ever receiving midazolam (**Panel A**), fentanyl (**Panel B**), dexmedetomidine (**Panel C**), and/or propofol (**Panel D**). Vertical lines highlight ICU (red) and health system-wide (green) innovations: *A.* Distribution of CAM-ICU cards to nursing staff; *B.* Lecture on Post-intensive Care Syndrome for ICU Providers; *C.* ‘ICU Delirium 101’ lecture for MICU nurses; *D.* ‘ICU Delirium 101’ lecture at UCLA Critical Care Symposium; *E.* UCLA Office of Health Informatics and Analytics (OHIA) approves development of EHR-based dataset to evaluate CAM-ICU and other delirium metrics. The *p*-value was calculated using Poisson regression, including sedation infusion administration data from all admissions involving mechanical ventilation.

The proportion of mechanically ventilated patients ever receiving continuous midazolam infusions declined from a peak of 86% in July 2013, and dropped to a nadir of 27% in November 2016 (widest absolute monthly difference = 60%; March 2013 to December 2016 monthly IRR = 0.99; *p* < 0.0001 for trend) (Figure 3). Conversely, the proportion of mechanically ventilated patients ever receiving dexmedetomidine and propofol infusions, respectively, rose from nadirs of 2% and 3% in March and July 2013, respectively, to peaks of 38% and 59% in September and May 2016 (widest differences = 36% and 56%, respectively; March 2013 to December 2016 monthly IRR = 1.02 and 1.02, respectively; both *p* < 0.0001) (Figure 2). There was no significant change in the administration of fentanyl infusions.

Additionally, regarding continuous sedative infusion dosing in mechanically ventilated patients, the cumulative midazolam milligrams infused declined from a peak mean (SD) of 920 (4675) in October 2013 to a nadir of 26 (67) in May 2014 (March 2013 to December 2016, monthly IRR = 0.97; *p* = 0.009). Conversely, the cumulative micrograms and milligrams of dexmedetomidine and propofol, respectively, rose from nadirs of 6 (26) and 421 (1177) in November and September 2013, respectively, to peaks of 1810 (5085) and 6178 (14,692) in March 2016 (monthly IRR = 1.03 [*p* < 0.0001] and 1.02 [*p* = 0.02], respectively). There was no significant change in cumulative fentanyl dosing.

## 4. Discussion

Driven by an interdisciplinary team and champions, this grassroots effort employed common strategies (e.g., one-time lectures) to fill key delirium knowledge gaps, resulting in robust improvements in daytime and nighttime CAM-ICU documentation. While primarily focused on delirium detection, this educational effort also yielded an unexpected improvement in sedation practices, specifically a reduction in the use of benzodiazepine infusions and a concomitant rise in non-benzodiazepine infusions, suggesting that interdisciplinary quality improvement projects can contribute to improvements in standard-of-care practices.

Although this educational project was performed nearly a decade ago, and we have no post-2016 MICU data to evaluate its sustainability, it is still highly relevant to modern-day (e.g., post-2018 PADIS and post-COVID-19), real-world ICU delirium practices worldwide, where persistent screening inconsistencies pose ongoing implementation challenges [9,20,21,22,23]. While e-learning and asynchronous delirium education tools may represent better options for in-person learning [24,25,26,27], this lecture-based approach may represent a more feasible and effective option in small and/or resource-limited (e.g., fewer stakeholders) settings, where a simple effort may be more familiar and sufficient to catalyze meaningful practice changes. Conversely, widely available and accessible technological resources could also expedite these types of projects, particularly in settings with constrained resources and lower nurse-to-patient ratios. For example, digital tools (e.g., virtual meetings, video-based education) could comprise e-learning or hybrid-learning models and pose more practical options for widespread education and awareness building. For ICUs embarking on the quality improvement initiative development process, we hope that this effort can highlight the importance of foundation-building via education regarding fundamentals, with a focus on more complex bedside actions thereafter. An inspired grassroots effort could then motivate champions and simple protocol changes (e.g., mandated daily documentation) and jumpstart larger delirium projects and delirium-focused culture shifts. Therefore, while our project occurred years ago and the results have aged, the project feasibility and robust improvements compelled us to share our strategy to benefit the many ICUs seeking basic and low-risk strategies to overcome standard-of-practice delirium inertia and jumpstart incremental changes when no other means are available and/or prior efforts have failed.

With champion-delivered lectures that focused on several ICU delirium topics, our effort generated intense interest from ICU staff and leadership that gained buy-in from health system administrators. This education motivated immediate improvements in CAM-ICU documentation, with improvements (>70%) reaching or exceeding those of other similar efforts (11% to 100%) [8]. Although “delirium: unable-to-assess” documentation rates paralleled those of prior studies, our monthly delirium incidence of 20–30% was lower than other quality improvement projects, possibly suggesting incorrect positive and negative CAM-ICU scoring despite improvements in documentation [8,20,28,29,30,31]. Nevertheless, a significant rise in documentation provided a starting point and foundation for future projects aimed at optimizing correct CAM-ICU documentation.

To our knowledge, this is among the first lecture-based, single-ICU quality improvement projects to yield such robust improvements in clinical behaviors. Previous efforts have attempted similar work on topics such as hand hygiene, but observed compliance differed across provider groups [32,33]. Meanwhile, inpatient delirium efforts involving in-person training, teaching modules, and care manuals found variable success in motivating sustained changes to care behaviors [22,34,35]. While our results are derived from a single academic MICU, our methodology, including leadership by an interdisciplinary champion-led team, could inform future similar delirium improvement efforts.

An essential component of this effort was the generation of an EHR dataset to affirm pre-project baseline behaviors and evaluate the effect, if any, of the project on key delirium-related variables. As the dataset was conceived after key components of the project had been delivered, we were unable to adopt audit-and-feedback techniques to further motivate adherence to delirium-friendly practices. While we presented EHR-produced performance data and improvements to bedside staff at divisional grand rounds in early 2016, it is unclear whether this presentation informed the durability or quality of provider behaviors moving forward. While strategies for delirium detection are widely available in the literature, there is little information on long-term sustainability after quality improvement project implementation [8]. As adherence often degrades without continued reinforcement, future efforts must embed a data-driven audit-and-feedback framework to maintain guideline-based delirium practices.

As a secondary topic of our education, we focused on sedation practices, including benzodiazepine infusions, as a potent risk factor for delirium, recommending the use of non-benzodiazepine alternatives such as dexmedetomidine and propofol [3,36]. As this topic encompassed only a few minutes of the education, we were surprised that our benzodiazepine infusion nadir of 27% mimicked those observed in sedation-focused efforts (e.g., ~22% in a formal protocol-based study) [37,38]. Whether the observed sedation trends were a byproduct of the educational effort, a response to previously non-documented delirium assessments, the result of unmeasured cultural changes in the ICU, or motivated by external guidelines (e.g., PADIS) is unclear, but represents an intriguing area of investigation. Future efforts should incorporate education on benzodiazepines, as it has the potential to create even a small amount of change.

Despite the feasibility of this project, several limitations warrant discussion. First, our effort is limited in its generalizability, given the single ICU design. However, the observed change in MICU practices highlights the profound effect of stakeholder, physician, and nurse buy-in along with education as facilitators of improved care behaviors. If lacking champions or facing barriers to in-person teaching, ICU administrators should evaluate existing delirium education frameworks (e.g., the “ICU Delirium Playbook” [26]) and consider the use of digital and/or contact-less platforms (i.e., teleconferencing, webinars, simulations) to provide necessary guideline-based education. Second, as much of this effort evolved organically after a new provider arrived and was made aware of ICU delirium knowledge gaps while providing care, no structured plan existed for this work. Given the unstructured project framework, we felt it more appropriate to examine crude trends than perform robust longitudinal analyses. Moreover, rigor can be added to inform and enhance future similar efforts, for example, by adding other clinically important delirium-related variables to the EHR-based dataset (e.g., patient mobility (e.g., rehabilitation staff interactions) and promotion of nighttime sleep (e.g., from nurse flowsheets or notes)). Though retrospective, our EHR-based dataset was invaluable in validating the impact of our efforts. Whether structured or impromptu, future delirium-based efforts must include informatics teams as a key partner in the implementation planning and tracking of important delirium management variables, such as early mobilization or sleep protocols. Third, it is unclear as to what degree the interdisciplinary effort influenced the observed practice changes, as opposed to landmark publications and guidelines [7]. However, significant knowledge gaps were present prior to the project, suggesting a lack of uptake of pre-existing literature, and the rapid rise in documentation and sedation practices after interdisciplinary effort initiation suggested minimal contribution of external forces, if any. Finally, as discussed above, we acknowledge that our educational project was performed a decade ago. However, given the past COVID-19 pandemic, it is likely that standard-of-care delirium practices regressed as a result of shifting critical care priorities. As the pandemic also witnessed a rise in ICU delirium and, for staff, increased burnout symptoms, which can be exacerbated when caring for a delirious patient, the setting is ripe for delirium education. Furthermore, as multiple ICUs all over the world currently report limited use of a validated assessment or call for improvement in delirium management, this study still shows relevance, particularly in ICUs that have never implemented a delirium effort or failed during prior attempts [21,23].

In conclusion, while work remains to optimize provider education regarding delirium and motivate guideline concordant projects, we demonstrate that a grassroots, rigorous lecture-based initiative, when delivered by experienced champions and an interdisciplinary team, can serve as a starting point and yield dramatic improvements in delirium documentation and sedation practices. Though performed several years ago, this study’s framework still applies today and can help ICUs seeking to narrow delirium education gaps and improve delirium-related outcomes.

## Figures and Tables

**Table 1:** “Delirium 101” lecture outline for ICU nursing staff.

Delirium Theme	Specific Topics
Epidemiology and Clinical Features	DSM-5 Definition: Acute disturbance of awareness, change from baseline cognition High prevalence in ICUs (up to 80% of mechanically ventilated patients) Hypoactive more common in ICU and harder to identify than hyperactive subtype
Associated Outcomes	Longer Duration of Mechanical Ventilation Longer ICU and Hospital Length of Stay Early Death Disabling Post-ICU Cognitive, Mental Health, Physical, and Mental Health Impairments High Costs ($150 billion in USA)
Potential Risk Factors	Older Age Sedative Infusions, including Benzodiazepines Medical Comorbidities Social Isolation Sleep-Wake Disturbance Immobility
Screening	Confusion Assessment Method for the ICU (CAM-ICU): Validated Bedside Tool Requiring <2 Minutes to Complete Feature 1—Acute Change or Fluctuating Course of Mental Status Feature 2—Inattention (Demonstration) Feature 3—RASS/Sedation Level (Demonstration) Feature 4—Disorganized Thinking (Demonstration)
Management and Prevention	Established Guidelines (Pain, Agitation, Delirium, Immobility, Sleep [PADIS]) Established Implementation Frameworks (ABCDEF Bundle) Prevention Begins with Detection Sedation Minimization; Favor Dexmedetomidine/Propofol Over Benzodiazepines Sleep-Wake Improvement Strategies Early Mobility Strategies

Abbreviations: ICU = intensive care unit; DSM-5 = *Diagnostic and Statistical Manual of Mental Disorders*, fifth edition; CAM-ICU = the Confusion Assessment Method for the Intensive Care Unit; PADIS = 2018 Clinical Practice Guidelines for the Prevention and Management of Pain, Agitation/Sedation, Delirium, Immobility, and Sleep Disruption in Adult Patients in the ICU; ABCDEF Bundle = Assess, Prevent, and Manage Pain, Both Spontaneous Awakening Trials (SAT) and Spontaneous Breathing Trials (SBT), Choice of Analgesia and Sedation, Delirium: Assess, Prevent and Manage, Early Mobility and Exercise, and Family Engagement and Empowerment Bundle.

**Table 2:** ICU patient demographic and clinical characteristics.

Characteristics	2013 ^a^	2014	2015	2016	Overall
Patients	789	1009	951	863	3612
Age, Median (IQR)	62 (49, 73)	61 (49, 72)	64 (50, 74)	62 (49, 72)	62 (49, 73)
Female, n (%)	379 (48%)	467 (46%)	416 (44%)	386 (45%)	1648 (45%)
Non-White Race, n (%)	279 (35%)	360 (36%)	368 (39%)	335 (39%)	1342 (37%)
Hispanic, n (%)	160 (20%)	207 (21%)	181 (19%)	174 (20%)	722 (20%)
ICU Admissions, n	941	1237	1177	1115	4470
ICU Length of Stay, Median (IQR) Days	5 (3, 9)	5 (3, 8)	5 (3, 8)	5 (3, 9)	5 (3, 8)
Hospital Length of Stay, Median (IQR) Days	11 (5, 24)	10 (5, 22)	10 (5, 21)	11 (5, 23)	10 (5, 22)
Required Mechanical Ventilation, n (%)	291 (37%)	384 (38%)	381 (40%)	392 (45%)	1448 (40%)
Mechanical Ventilation Days, Median (IQR) ^b^	4 (2, 8)	3 (2, 7)	4 (2, 9)	3 (2, 8)	3 (2, 7)
Received Sedative Infusion, n (%) ^b^					
Midazolam	216 (69%)	249 (58%)	196 (47%)	185 (41%)	846 (52%)
Dexmedetomidine	29 (9%)	73 (17%)	88 (21%)	123 (27%)	313 (19%)
Fentanyl	222 (71%)	281 (65%)	273 (65%)	289 (64%)	1065 (66%)
Propofol	54 (17%)	122 (28%)	148 (35%)	198 (44%)	522 (32%)

Abbreviations: ICU = intensive care unit; IQR = interquartile range; ^a^ Excludes January and February 2013; Electronic Health Record introduced on 1 March 2013; ^b^ Including all admissions requiring mechanical ventilation (N = 1616).

## Data Availability

The datasets generated during and/or analyzed during the current study are available from the corresponding author on reasonable request.

## Public Involvement Statement

No public involvement in any aspect of this research.

## Guidelines and Standards Statement

This analysis was reported in accordance with SQUIRE 2.0 guidelines [16].
